# Choroidal thickness in Waardenburg syndrome

**DOI:** 10.3205/oc000111

**Published:** 2019-06-18

**Authors:** Pukhraj Rishi, Priyansha Multani, Vishnu Vahan Prasan, Ekta Rishi, Yamini Attiku

**Affiliations:** 1 Shri Bhagwan Mahavir Vitreoretinal Services, Sankara Nethralaya, Chennai, India

**Keywords:** eye, pigmentation, Waardenburg syndrome, heterochromia, choroidal hypopigmentation, choroidal thickness, optical coherence tomography

## Abstract

**Purpose:** To assess the choroidal thickness in differently pigmented areas of the fundus in a 46-year-old female with Waardenburg syndrome.

**Methods:** Retrospective, case review. Choroidal thickness was measured using swept-source optical coherence tomography (SS-OCT, Topcon DRI OCT-1 Atlantis) and compared between the pigmented and hypopigmented areas within the same eye and between the two eyes.

**Results:** Best corrected visual acuity (BCVA) was 20/20 in both eyes. The right fundus had a variegated appearance without choroidal hypopigmentation. The left fundus had choroidal hypopigmentation beyond the superotemporal arcade up to the periphery. Subfoveal choroidal thickness was 455 µ in the right eye and 569 µ in the left eye. In the left eye, the comparison of two equidistant points from the foveola along a radial scan passing through the superotemporal hypopigmented area revealed a thinner choroidal thickness (457 µ) compared to the corresponding point in the pigmented inferonasal quadrant (591 µ).

**Conclusion:** Choroidal thickness is decreased in the hypopigmented area of the fundus compared to the pigmented area in subjects with Waardenburg syndrome. The overall thickness of the choroid in such eyes could still be more than the mean value in the normal population.

## Introduction

The Waardenburg syndrome is a rare, dominantly inherited auditory-pigmentary syndrome with congenital deafness, dystopia of the medial canthus, telecanthus, synophrys, broadening of nasal root, and pigmentary disorder of the irides, skin and hair [1]. It has myriad clinical features with incomplete penetrance and variable expressivity, even among members of the same family. Pigmentary abnormalities of fundus coloration are an integral part of this syndrome and can simulate other conditions, especially sector ocular melanocytosis [1], [2].

The swept source optical coherence tomography (SS-OCT) uses a novel fourier domain detection method utilizing a tunable dye laser of longer wavelength in 1 micron range, allowing for deeper light penetration through the choroidal tissue [3]. It facilitates better visualization and characterization of choroidal pathology, especially in darkly pigmented races. Hereby, we report a case of a 46-year-old female with fundus pigmentary abnormalities who was referred for further evaluation. SS-OCT was used to assess the choroidal thickness in different pigmentary areas of the fundus.

## Case description

**Figure 1:**
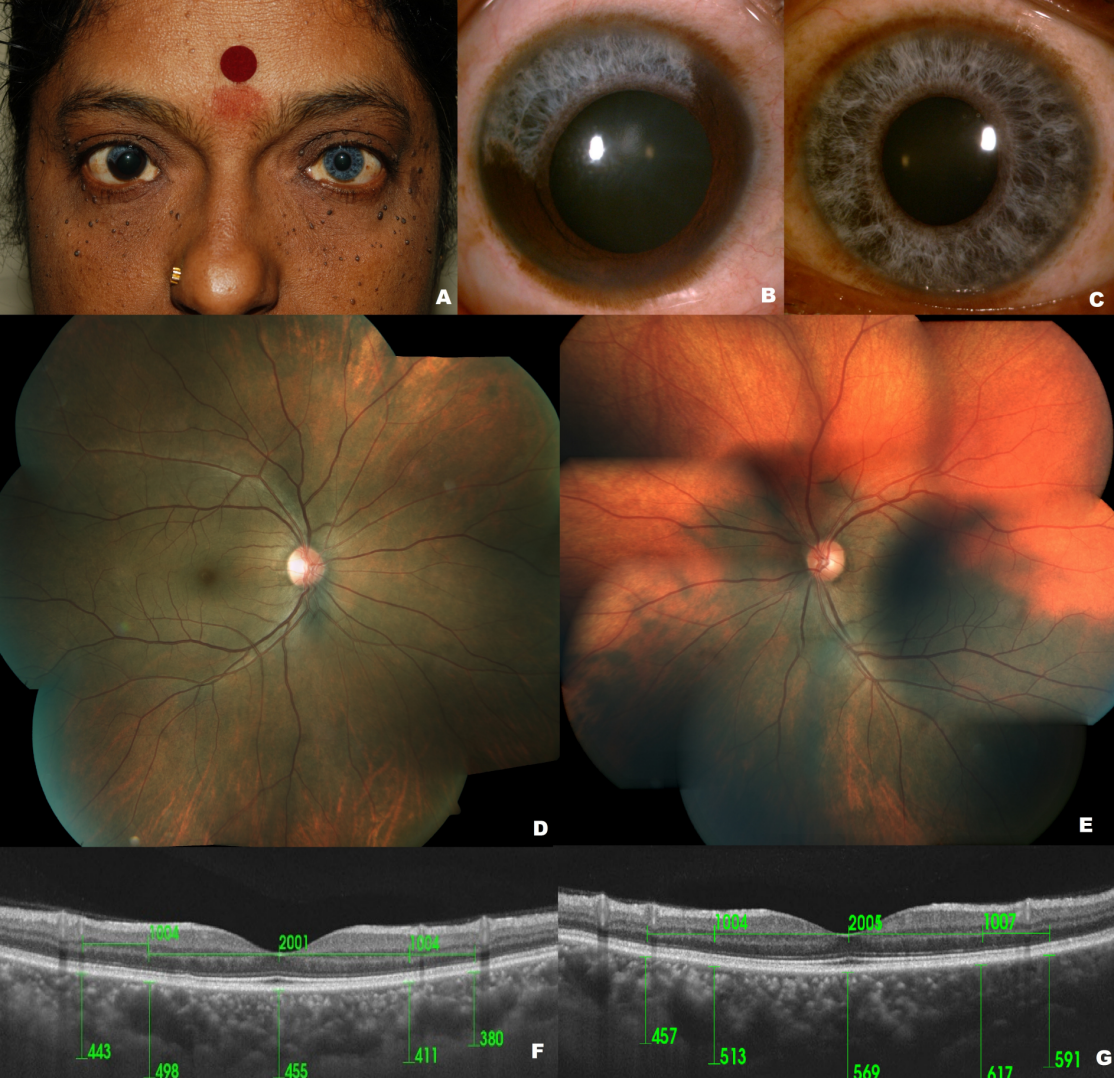
External photograph (A) reveals telecanthus, flare of the medial eyebrows and hypoplastic nasal alae. Slit lamp image of the right eye (B) reveals a sectoral hypopigmentation of the iris extending from 9’ o’clock meridian to 1’30” o’clock meridian. Slit lamp image of the left eye (C) reveals a complete hypopigmentation of the iris imparting it a ‘brilliant blue’ appearance. Color fundus montage of the right eye (D) reveals no area of hypopigmentation. Color fundus montage of the left eye (E) reveals a partially hypopigmented area in the superior fundus. SS-OCT of the right eye (F) reveals a subfoveal choroidal thickness of 455 µ. SS-OCT of the left eye (G) reveals a subfoveal choroidal thickness of 569 µ, a choroidal thickness of 591 µ in the pigmented fundus, and a choroidal thickness of 457 µ in the hypopigmented fundus.

A 46-year-old Indian female presented with watering of the right eye since 5 months. History revealed a premature graying of the hair and ‘blue’ color of the eyes since childhood. She also reported her son having similar color of the eyes. However, he was not available for examination. The rest of the family history was non-contributory. She denied any history of hearing loss. The best corrected visual acuity (BCVA) was 20/20 in both eyes. Gross examination revealed telecanthus, flare of the medial eyebrows, and hypoplastic nasal alae (Figure 1A (Fig. 1)). Symmetrical pigmented papillomatous lesions on face, neck, and shoulders and pigmented nevi of the skin temporal to the lateral canthus of the left eye were noted. Anterior segment examination revealed a sectoral heterochromia iridum in the right eye with a hypopigmented ‘brilliant blue’ iris extending for 4 hours, superotemporally (Figure 1B (Fig. 1)). Iris stromal and pupillary ruff atrophy and poor pupillary dilation in the affected sector were noted. The left eye had a ‘brilliant blue’ iris with relatively poor pupillary dilation (Figure 1C (Fig. 1)). The right fundus had a variegated appearance without choroidal hypopigmentation (Figure 1D (Fig. 1)). The left fundus had choroidal hypopigmentation beyond the superotemporal arcade up to the periphery (Figure 1E (Fig. 1)). There was no evidence of vitiligo, scleral pigmentation, or any sign of chronic uveitis in both eyes. Based on the clinical findings, she was diagnosed of Waardenburg syndrome. The choroidal thickness was measured using swept-source optical coherence tomography (SS-OCT, Topcon DRI OCT -1 Atlantis) and compared between the two eyes and between the pigmented and the vitiliginous areas in the left eye. The subfoveal choroidal thickness was 455 µ (Figure 1F (Fig. 1)) and 569 µ in the right and left eye, respectively (Figure 1G (Fig. 1)). In the left eye, a comparison of two equidistant points from the foveola along a radial scan passing through the superotemporal vitiliginous area revealed a thinner choroid (457 µ) compared to the corresponding point in the pigmented inferonasal quadrdant (591 µ).

## Discussion

There is a highly variable expression of traits in the Waardenburg syndrome. This heterogeneity can even be observed among members of the same family. So, fundus pigmentary abnormalities seen in isolation could confound an ophthalmologist. Various disorders can present with disturbances in choroidal pigmentation. Conditions presenting with choroidal hypopigmentation include ocular albinism, choroidal vitiligo, Vogt-Koyanagi-Harada syndrome, high myopia, and Waardenburg syndrome. Disorders with increased choroidal pigmentation are isolated ocular melanocytosis, oculo(dermal) melanocytosis, giant choroidal nevus, diffuse choroidal melanoma, and bilateral diffuse uveal melanocytic proliferation (BDUMP). The ocular melanocytosis simulates the Waardenburg syndrome the most. Both are congenital and can be associated with iris heterochromia. However, in oculo(dermal) melanocytosis, other features such as episceral/scleral pigmentation and iris mamillations may be present. It is critical to distinguish between these two distinct clinical entities as oculo(dermal) melanocytosis, even if sectoral, is associated with the risk of development of choroidal melanoma in the area of increased choroidal pigmentation and necessitates life-long follow-up [4]. In contrast, the Waardenburg syndrome has no association with malignancy. 

Goldberg described that iris and fundus pigment abnormalities tend to parallel each other and are an integral part of this syndrome [2]. In 1978, Delleman and Hageman concluded that 10 of the 15 patients in their study who had iris pigmentary abnormalities had corresponding findings in their fundus as well [5]. In our patient, the striking facial features, iris heterochromia, ‘brilliant blue’ iris, and choroidal hypopigmentation were suggestive of Waardenburg syndrome but the pigmentation pattern of iris and fundus did not match.

The choroidal thickness was less in the hypopigmentated area of the left fundus compared to the pigmented area. Subfoveal choroidal thickness was greater than that reported in the general population, again probably because of pigmentation [6]. The normal subfoveal choroidal thickness in Indian eyes on SS-OCT has been reported to be 307±79 µm [7]. Shields et al. have reported a slightly lesser choroidal thickness (mean 197 µ) on OCT imaging of the hypopigmented area compared to normal choroid in the fellow eye (mean 243 µ), but the choroidal thickness within the same eye at different areas were not compared [8]. Unlike the observations in our case where the subfoveal choroidal thickness was much higher than normal values, choroidal thickness in their series was much closer to the normative data. This could be related to racial differences. On the contrary, Choudhry et al. have reported a thicker hypopigmented foveal choroid (430 µ and 435 µ) in their case report [9]. Histological examination of the hypopigmented iris has shown a significant decrease in melanosome size. Defects in melanosome migration and melanin synthesis have been implicated [10]. Dense hyperpigmentation next to a hypopigmented area may be attributed to defective migration of melanocytes leading to their increased concentration in localized area of the choroid and their relative absence in other areas leading to hypopigmentation. This is in contrast to a more uniform pigmentation of the choroid if the melanosomes were to have migrated normally and distributed evenly [6]. Histological findings of the fundus appearance in this syndrome have not been described. This variegated pattern of fundus pigmentation and varying choroidal thickness could be attributed to the same developmental anomaly leading to a cutaneous manifestation of piebaldism.

## Notes

### Competing interests

The authors declare that they have no competing interests.
